# Supplementary material for: Bi-terminal fusion of intrinsically-disordered mussel foot protein fragments boosts mechanical strength for protein fibers
Source: Nat Commun. 2023 Apr 14;14:2127. doi: 10.1038/s41467-023-37563-0 (PMC10104820; doi:10.1038/s41467-023-37563-0)
Supplement: Supplementary file 1 — Supplementary Information [file 41467_2023_37563_MOESM1_ESM.pdf]

Supplementary information

Bi-terminal Fusion of Intrinsically-Disordered Mussel Foot Protein Fragments Boosts Mechanical Strength for Protein Fibers

Authors: Jingyao Li[1], Bojing Jiang[1], Xinyuan Chang[1], Han Yu[1], Yichao Han[1], Fuzhong Zhang[1,2,3]

Affiliations:

[1]Department of Energy, Environmental and Chemical Engineering, Washington University in St. Louis, One Brookings Drive, Saint Louis, MO 63130, USA

[2]Division of Biological & Biomedical Sciences, Washington University in St. Louis, One Brookings Drive, Saint Louis, MO 63130, USA

[3]Institute of Materials Science & Engineering, Washington University in St. Louis, One Brookings Drive, Saint Louis, MO 63130, USA

Correspondence should be addressed to F.Z. (email: fzhang@seas.wustl.edu).

This file includes:

Supplementary Table 1 to 18

Supplementary Figure 1 to 22

Supplementary note for protein yield estimation

Supplementary Table 1. Amino acid sequences of protein segments used in this study.

	Sequence	Reference
1xFGAILSS tandem repeat	GRGGLGGQGAGFGAILSSGGAGQGGYG<br>GLGSQGTS	Li et al.[1]
1xKLVFFAE tandem repeat	GRGGLGGQGAGKLVFFAEGGAGQGGY<br>GGLGSQGTS	Li et al.[1]
Titin (4xTitin)	PPFFDLKPVSVDLALGESGTFKCHVTGT<br>APIKITWAKDNREIRPGGNYKMTLVENT<br>ATLTVLKVTKGDAGQYTCYASNVAGK<br>DSCSAQLGVQEPPRFIKKLEPSRIVKQDE<br>HTRYECKIGGSPEIKVLWYKDETEIQESS<br>KFRMSFVESVAVLEMYNLSVEDSGDYT<br>CEAHNAAGSASSSTSLKVKEPPVFRKKP<br>HPVETLKGADVHLECELQGTPPFQVSW<br>HKDKRELRSGKKYKIMSENFLTSIHILNV<br>DSADIGEYQCKASNDVGSDTCVGSITLK<br>APPRFVKKLSDISTVVGEEVQLQATIEGA<br>EPISVAWFKDKGEIVRESDNIWISYSENI<br>ATLQFSRAEPANAGKYTCQIKNEAGTQE<br>CFATLSVLE	Bowen et al.[2]
GFP	SKGEELFTGVVPILVELDGDVNGHKFSV<br>SGEGEGDATYGKLTLKFICTTGKLPVPW<br>PTLVTTFAYGLQCFARYPDHMKQHDFF<br>KSAMPEGYVQERTIFFKDDGNYKTRAE<br>VKFEGDTLVNRIELKGIDFKEDGNILGH<br>KLEYNYNSHNVYIMADKQKNGIKVNFK<br>IRHNIEDGSVQLADHYQQNTPIGDGPVL<br>LPDNHYLSTQSALSKDPNEKRDHMVLL<br>EFVTAAGITHGMDELYK	Han et al.[3]
SH3	VQISTLFEALYDYEARTEDDLSFHKGEK<br>FQILNSSEGDWWEARSLTTGETGYIPSN<br>YVAPVDRLDYKDDDDK	Zhang et al.[4]
^{N}Mfp5 (^{N}M)	SEEYKGGYYPGNTYHYHSGGSYHGSGY<br>HGGYKGKYY	This work
^{N}Mfp5(YtoS)	SEESKGGSSPGNTSHSHSGGSSHGSGSH<br>GGSKGKSS	This work
^{C}Mfp5 (^{C}M)	KAKKYYYKYKNSGKYKYLKKARKYHR<br>KGYKKYYGGGSS	This work
^{C}Mfp5(YtoS)	KAKKSSSKSKNSGKSKSLKKARKSHRK<br>GSKKSSGGGSS	This work
^{C}Mfp5(KRtoS)	SASSYYYSYSNSGSYSYLSSASSYHSSGY<br>SSYYGGGSS	This work
His-Tag	HHHHHHHHHH	Li et al.

Supplementary Table 2. Summary of mechanical properties of all ^{N}M-16xFGA-^{C}M fibers tested in this study.

	Ultimate Tensile Stress (MPa)	Young's Modulus (GPa)	Strain at Break (%)	Toughness (MJ*m^{-3})	Diameter (mm)
1	358	2.6	38	100	20
2	387	2.8	59	160	19
3	393	3.0	42	100	20
4	416	3.1	43	110	19
5	373	2.5	47	120	20
6	419	3.8	43	120	19
7	399	3.4	44	100	19
8	385	2.4	47	150	20
9	468	3.1	42	110	18
10	465	2.9	42	110	18
Average	406	3.0	45	118	19
Standard Deviation	36	0.44	5.7	21	0.70

Supplementary Table 3. Summary of mechanical properties of all 16xFGA-M fibers tested in this study.

	Ultimate Tensile Stress (MPa)	Young's Modulus (GPa)	Strain at Break (%)	Toughness ($MJ*m^{-3}$)	Diameter (mm)
1	267	3.8	54	120	31
2	288	2.7	67	150	30
3	297	3.3	57	140	29
4	277	3.8	64	150	31
5	275	3.6	51	120	30
6	264	2.7	44	90	30
7	251	2.7	36	70	30
8	271	4.0	60	130	30
9	287	4.3	61	140	30
10	286	4.2	52	120	29
Average	276	3.5	55	123	30
Standard Deviation	14	0.61	9.5	26	0.51

Supplementary Table 4. Summary of mechanical properties of all ^{N}M(YtoS)-16xFGA-^{C}M fibers tested in this study.

	Ultimate Tensile Stress (MPa)	Young's Modulus (GPa)	Strain at Break (%)	Toughness ($MJ*m^{-3}$)	Diameter (mm)
1	250	3.0	52	100	42
2	249	3.4	73	140	43
3	279	3.6	79	180	41
4	248	3.4	50	100	41
5	248	2.3	49	100	42
6	227	2.9	50	90	42
7	290	3.4	66	150	40
8	265	3.2	57	110	41
9	278	2.3	44	90	39
Average	259	3.1	58	118	41
Standard Deviation	20	0.49	12	32	1.1

Supplementary Table 5. Summary of mechanical properties of all ^{N}M-16xFGA-^{C}M(YtoS) fibers tested in this study.

	Ultimate Tensile Stress (MPa)	Young's Modulus (GPa)	Strain at Break (%)	Toughness (MJ*m^{-3})	Diameter (mm)
1	365	2.7	42	110	22
2	392	4.0	40	130	22
3	408	3.1	41	120	21
4	405	3.2	44	130	21
5	383	3.5	43	110	22
6	412	3.2	44	130	21
7	275	3.3	30	100	21
8	371	2.9	38	110	22
9	452	3.7	44	130	19
10	471	3.2	42	140	19
Average	406	3.3	41	121	21
Standard Deviation	33	0.38	4.1	13	1.2

Supplementary Table 6. Summary of mechanical properties of all ^{N}M-16xFGA-^{C}M(KRtoS) fibers tested in this study.

	Ultimate Tensile Stress (MPa)	Young's Modulus (GPa)	Strain at Break (%)	Toughness (MJ*m^{-3})	Diameter (mm)
1	349	2.6	52	130	36
2	469	1.9	41	120	29
3	475	1.9	41	100	29
4	368	2.2	56	140	31
5	391	3.4	41	130	31
6	404	3.1	49	140	31
7	512	3.4	43	130	29
8	405	3.3	55	150	31
9	376	3.0	51	140	31
10	493	2.3	43	120	29
Average	424	2.7	47	130	31
Standard Deviation	58	0.61	6.1	14	2.3

Supplementary Table 7. Summary of mechanical properties of all ^{C}M-16xFGA-^{C}M fibers tested in this study.

	Ultimate Tensile Stress (MPa)	Young's Modulus (GPa)	Strain at Break (%)	Toughness (MJ*m^{-3})	Diameter (mm)
1	409	4.0	26	60	15
2	451	3.7	33	90	16
3	414	3.3	38	100	16
4	441	3.9	32	100	16
5	484	4.1	31	100	15
6	408	2.9	33	80	17
7	391	2.9	47	130	17
8	349	2.8	30	80	16
Average	418	3.5	34	92	16
Standard Deviation	41	0.54	6.3	21	0.83

Supplementary Table 8. Summary of mechanical properties of all ^{N}M-16xFGA-^{C}M(YtoS) fibers spun in pH = 5.5 tested in this study.

	Ultimate Tensile Stress (MPa)	Young's Modulus (GPa)	Strain at Break (%)	Toughness (MJ*m^{-3})	Diameter (mm)
1	540	3.6	42	140	22
2	434	2.8	36	160	22
3	489	3.0	54	190	23
4	490	2.3	65	240	24
5	457	3.5	59	210	24
6	506	5.0	47	160	24
7	448	2.9	39	110	23
8	500	3.9	48	170	24
9	465	3.0	62	220	25
10	484	3.6	55	190	24
Average	481	3.4	51	179	23
Standard Deviation	31	0.74	10	39	1.1

Supplementary Table 9. Summary of mechanical properties of all ^{N}M-16xFGA-^{C}M(YtoS) fibers spun in pH = 11 tested in this study.

	Ultimate Tensile Stress (MPa)	Young's Modulus (GPa)	Strain at Break (%)	Toughness (MJ*m^{-3})	Diameter (mm)
1	399	2.9	43	110	26
2	364	2.1	49	130	27
3	363	2.3	40	90	27
4	333	2.8	39	80	28
5	389	2.5	46	130	26
6	336	2.9	43	100	28
7	360	4.1	41	110	28
8	374	3.2	43	110	27
9	359	2.8	40	90	27
10	398	4.0	43	120	26
Average	367	3.0	43	107	27
Standard Deviation	23	0.67	2.9	17	0.74

Supplementary Table 10. Summary of mechanical properties of all ^{N}M-16xKLV-^{C}M fibers tested in this study.

	Ultimate Tensile Stress (MPa)	Young's Modulus (GPa)	Strain at Break (%)	Toughness (MJ*m^{-3})	Diameter (mm)
1	529	5.3	27	100	16
2	444	5.0	28	90	18
3	449	3.9	26	70	16
4	432	2.9	46	210	18
5	441	4.6	31	90	18
6	444	3.8	26	80	18
7	452	5.0	45	160	18
8	452	3.8	34	110	18
9	403	3.8	30	80	19
10	494	4.2	29	180	16
Average	454	4.2	32	117	17
Standard Deviation	35	0.73	7.5	49	1.0

Supplementary Table 11. Summary of mechanical properties of all methanol-spun Titin fibers tested in this study.

	Ultimate Tensile Stress (MPa)	Young's Modulus (GPa)	Strain at Break (%)	Toughness ($MJ*m^{-3}$)	Diameter (mm)
1	91	0.57	133	90	52
2	99	1.7	137	110	52
3	98	1.4	151	120	51
4	92	1.7	122	90	51
5	106	1.7	155	130	49
6	81	1.3	77	50	51
Average	94	1.4	129	98	51
Standard Deviation	8.5	0.44	28	29	1.0

Supplementary Table 12. Summary of mechanical properties of all ^{N}M-Titin-^{C}M fibers tested in this study.

	Ultimate Tensile Stress (MPa)	Young's Modulus (GPa)	Strain at Break (%)	Toughness (MJ*m^{-3})	Diameter (mm)
1	235	1.5	39	80	25
2	236	3.3	67	120	28
3	212	2.6	36	80	26
4	222	2.0	41	80	27
5	206	2.3	54	90	28
6	206	2.0	54	90	28
7	243	2.7	31	70	25
8	210	2.5	44	70	27
9	272	1.4	53	90	28
10	211	1.4	43	80	30
Average	225	2.2	46	85	27
Standard Deviation	21	0.65	11	14	1.7

Supplementary Table 13. Summary of mechanical properties of all ^{N}M-16xAAA-^{C}M fibers tested in this study.

	Ultimate Tensile Stress (MPa)	Young's Modulus (GPa)	Strain at Break (%)	Toughness (MJ*m^{-3})	Diameter (mm)
1	140	2.8	95	120	25
2	142	2.5	81	100	26
3	151	2.6	139	180	27
4	145	1.9	118	150	29
5	141	2.6	121	150	29
6	122	2.5	109	120	27
7	137	2.9	139	160	29
8	130	2.5	129	150	30
Average	139	2.5	116	141	28
Standard Deviation	9.0	0.29	21	26	1.7

Supplementary Table 14. Summary of mechanical properties of all GFP fibers tested in this study.

	Ultimate Tensile Stress (MPa)	Young's Modulus (GPa)	Strain at Break (%)	Toughness (MJ*m^{-3})	Diameter (mm)
1	64	1.9	7.4	5.0	44
2	82	1.7	8.6	6.0	40
3	73	1.9	8.5	5.0	40
4	88	2.1	6.6	7.0	40
5	77	1.8	8.8	5.0	42
6	92	2.3	11	7.0	39
7	79	1.9	7.6	6.0	42
8	95	2.6	5.2	3.0	38
9	94	2.8	6.0	4.0	38
10	75	1.8	7.3	4.0	42
Average	82	2.1	7.7	5.2	41
Standard Deviation	10	0.38	1.5	1.3	2.0

Supplementary Table 15. Summary of mechanical properties of all ^{N}M-GFP-^{C}M fibers tested in this study.

	Ultimate Tensile Stress (MPa)	Young's Modulus (GPa)	Strain at Break (%)	Toughness ($MJ*m^{-3}$)	Diameter (mm)
1	122	1.9	119	120	26
2	122	2.4	91	110	26
3	109	2.1	87	80	28
4	131	3.1	80	100	25
5	122	2.5	87	100	26
6	111	2.2	97	100	28
7	131	3.2	76	90	25
8	169	1.7	72	100	25
Average	127	2.4	89	100	26
Standard Deviation	19	0.52	15	12	1.1

Supplementary Table 16. Summary of mechanical properties of all SH3 fibers tested in this study.

	Ultimate Tensile Stress (MPa)	Young's Modulus (GPa)	Strain at Break (%)	Toughness ($MJ*m^{-3}$)	Diameter (mm)
1	40	1.3	3.9	1.0	75
2	49	1.4	4.6	1.4	73
3	72	1.0	9.3	4.0	70
4	72	1.5	11	5.0	75
5	26	0.75	5.3	0.90	78
6	76	0.72	14	6.0	73
Average	56	1.1	7.9	3.0	74
Standard Deviation	21	0.35	3.9	2.2	2.9

Supplementary Table 17. Summary of mechanical properties of all ^{N}M-SH3-^{C}M fibers tested in this study.

	Ultimate Tensile Stress (MPa)	Young's Modulus (GPa)	Strain at Break (%)	Toughness ($MJ*m^{-3}$)	Diameter (mm)
1	284	2.9	38	70	22
2	219	2.1	39	60	25
3	256	3.0	30	50	22
4	298	2.8	34	70	21
5	264	2.5	32	60	22
6	247	3.0	32	50	23
7	228	1.8	45	70	25
8	199	2.7	28	40	26
Average	249	2.6	35	59	23
Standard Deviation	33	0.45	5.6	11	1.7

Supplementary Table 18. Examples of recombinant fiber protein titer and fiber strength.

Expression host	Protein homologue and origin species	Protein size (kDa)	Maximum reported yield (g/L)	Tensile strength (MPa)	Reference
E. coli	Rational design	58	8.0	483	This work
E. coli	Rational design	33	9.0	131	Arndt et al. [5]
E. coli	*E. australis,* chimeric	33	14.5	100	Schmuck et al. [6]
E. coli	*T. clavipes*	285	0.50	508	Xia et al.[7]
E. coli	*T. clavipes*	556	N.A.	1030	Bowen et al. [8]
E. coli	Rational design	146	N.A.	630	Li et al.[9]
E. coli	*A. diadematus*	76	N.A.	370	Heidebretch et al.[10]
P. pastoris	*T. clavipes*	65	0.66	N.A.	Fahnestock et al. [11]
S. cerevisiae	*T. clavipes*	94	0.45	N.A.	Sidoruk et al. [12]
S. frugiperda	*A. diadematus*	60	0.05	N.A.	Ittah et al. [13]
Baby hamster kidney cells	*A. diadematus*	60	0.05	100-200	Lazaris et al. [14]

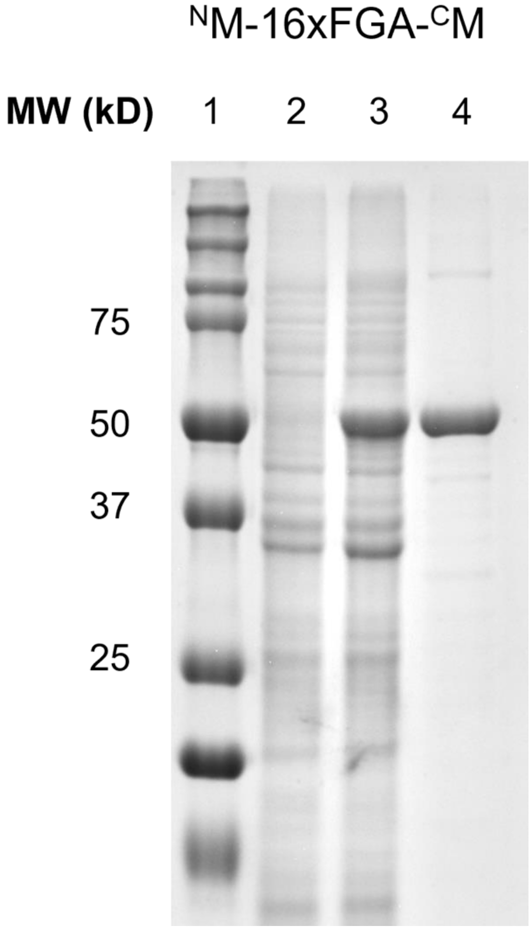

Supplementary Figure 1. Coomassie blue-stained 10% SDS-PAGE gel of E. coli whole cell lysate and purified ^{N}M-16xFGA-^{C}M protein. Lane 1, MW marker; lane 2, E. coli whole cell lysate before induction; lane 3, whole cell lysate after induction; lane 4, Ni-NTA column purified ^{N}M-16xFGA-^{C}M proteins. Source data are provided as a Source Data file.

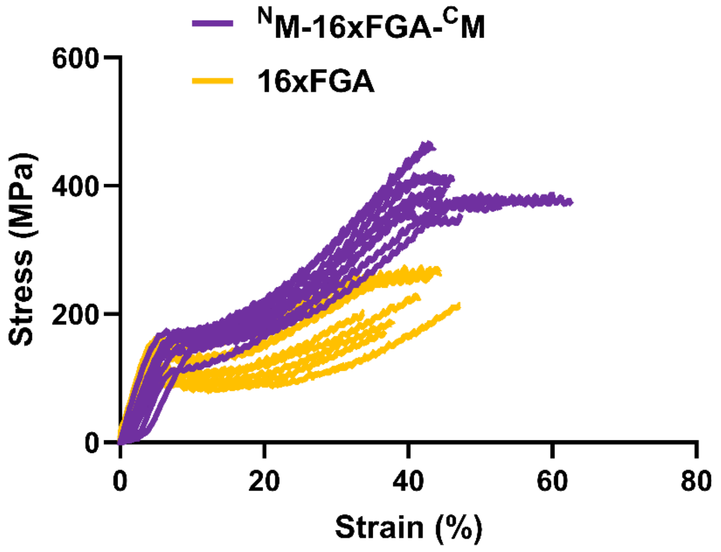

Supplementary Figure 2. Stress-strain curves of 16xFGA and ^{N}M-16xFGA-^{C}M fibers. Source data are provided as a Source Data file. 16xFGA mechanical data reproduced from previous publication (reproduced with permission,[1] Copyright 2021 American Chemical Society) serve as a comparison.

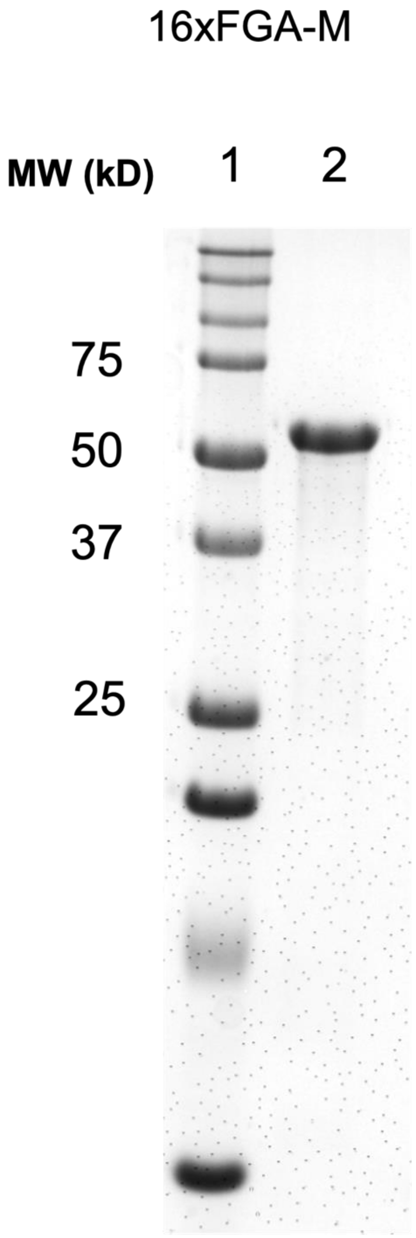

Supplementary Figure 3. Coomassie blue-stained 10% SDS-PAGE gel of purified 16xFGA-M protein. Source data are provided as a Source Data file.

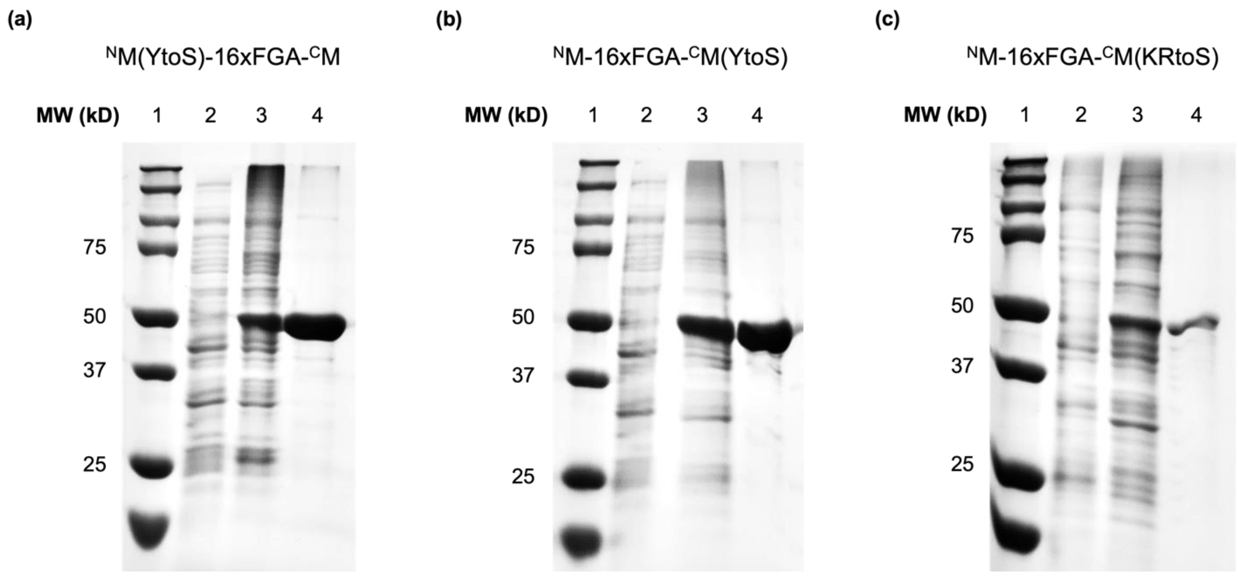

Supplementary Figure 4. Coomassie blue-stained 10% SDS-PAGE gel of E. coli whole cell lysate and purified proteins. Lane 1, MW marker; lane 2, E. coli whole cell lysate before induction; lane 3, whole cell lysate after induction; lane 4, Ni-NTA column purified proteins. Source data are provided as a Source Data file.

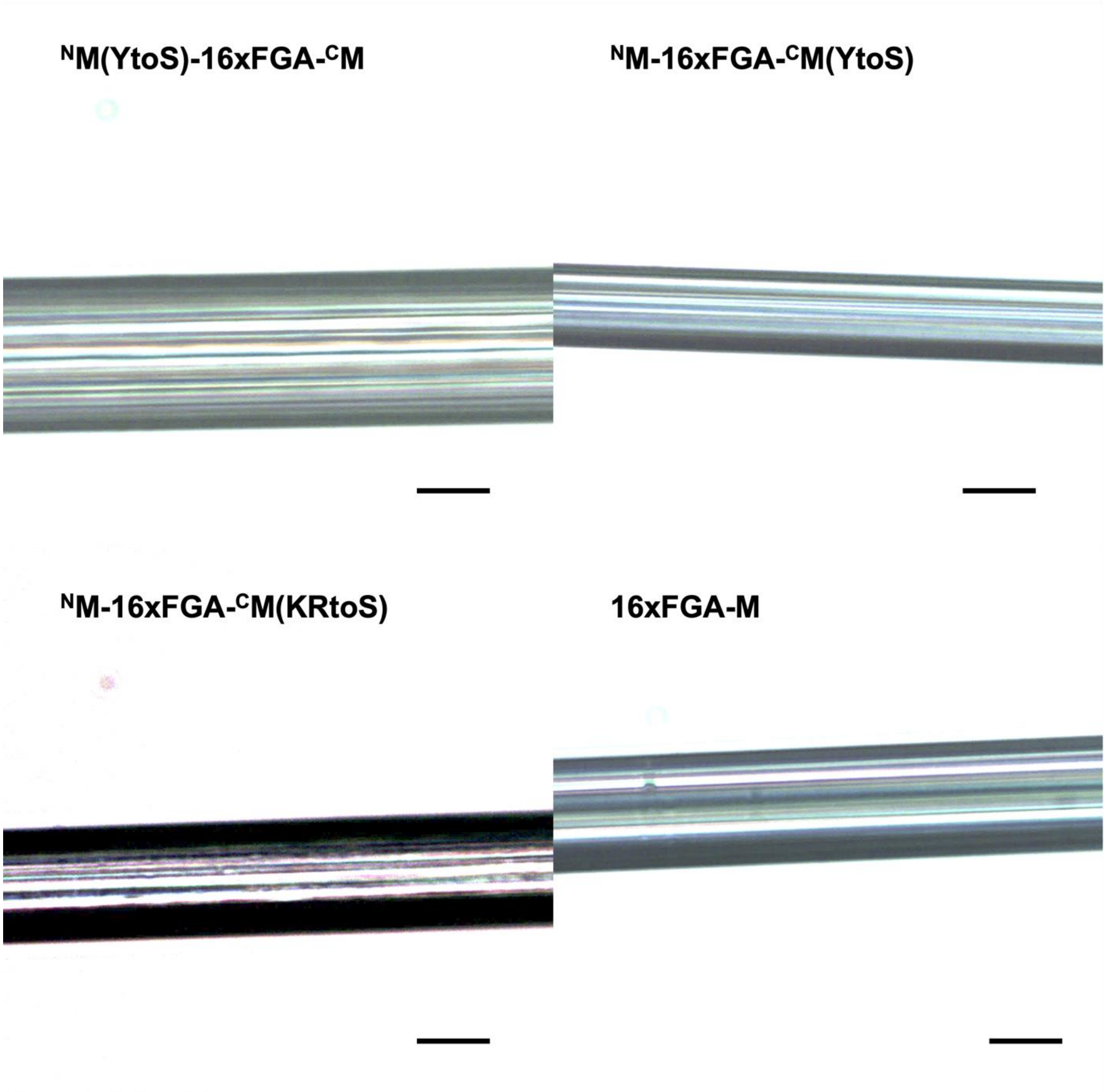

Supplementary Figure 5. Optical microscope images of btMfp5-fused 16xFGA variant fibers and 16xFGA-M fiber. Scale bars indicate 20μm.

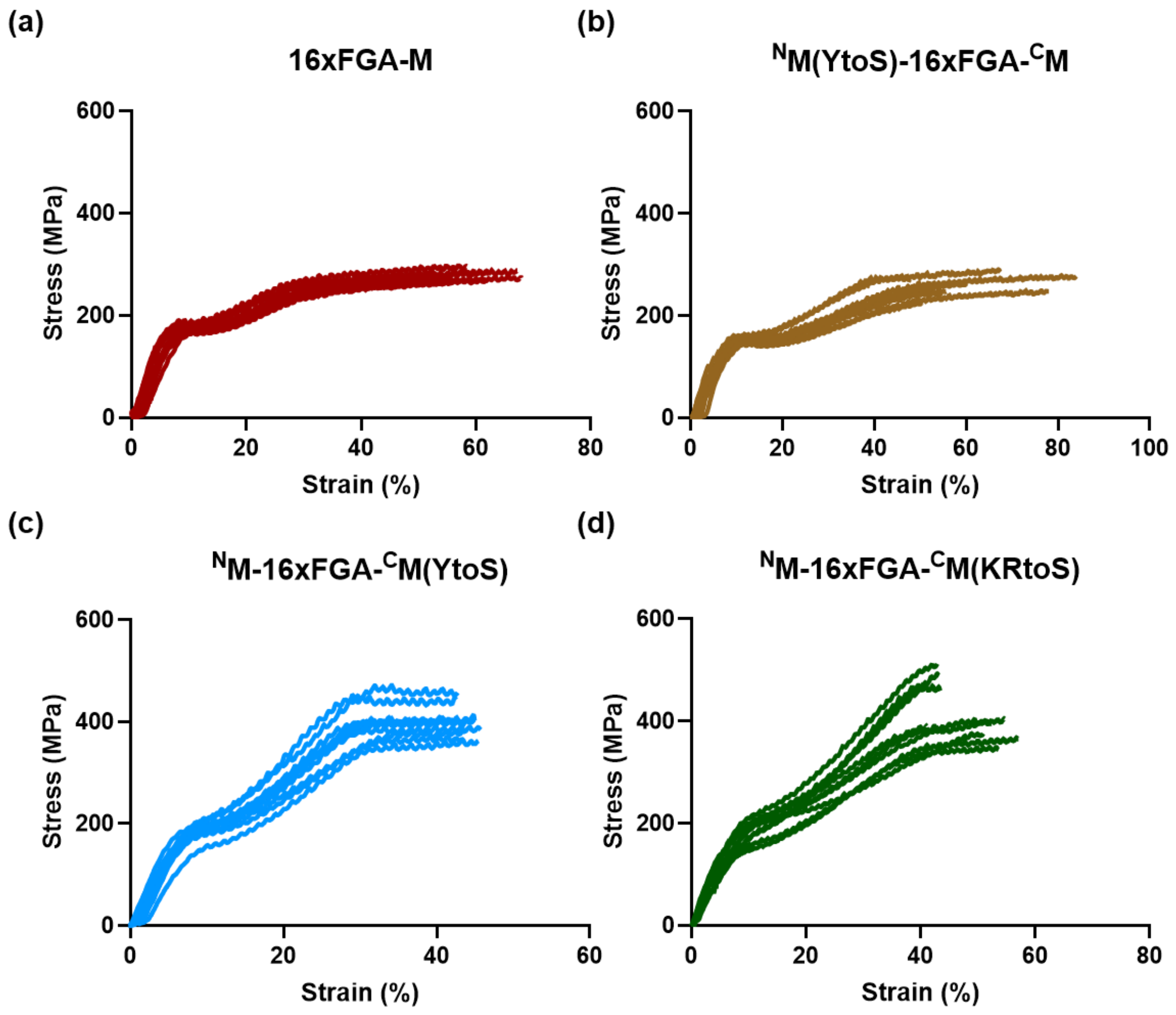

Supplementary Figure 6. Stress-strain curves of 16xFGA-M and btMfp5-fused 16xFGA variant fibers. Source data are provided as a Source Data file.

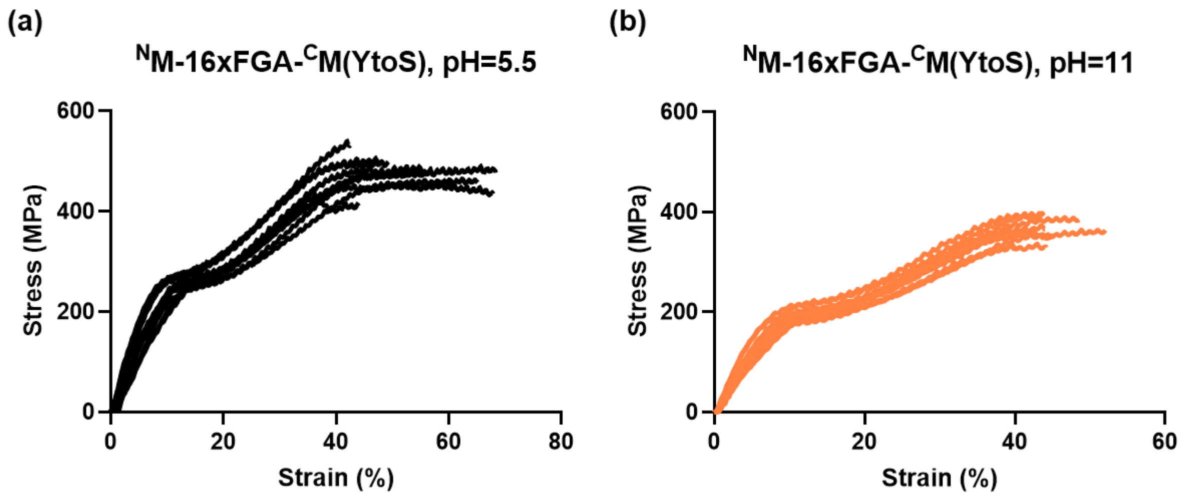

Supplementary Figure 7. Stress-strain curves of ^{N}M-16xFGA-^{C}M(YtoS) fibers spun in different pH in 95% methanol. Source data are provided as a Source Data file.

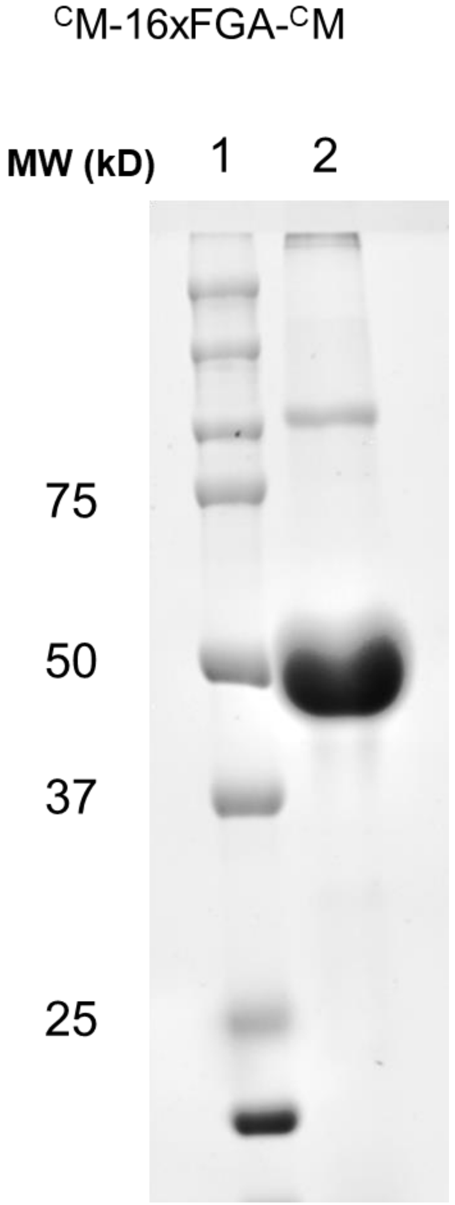

Supplementary Figure 8. Coomassie blue-stained 10% SDS-PAGE gel of purified ^{C}M-16xFGA-^{C}M protein. Source data are provided as a Source Data file.

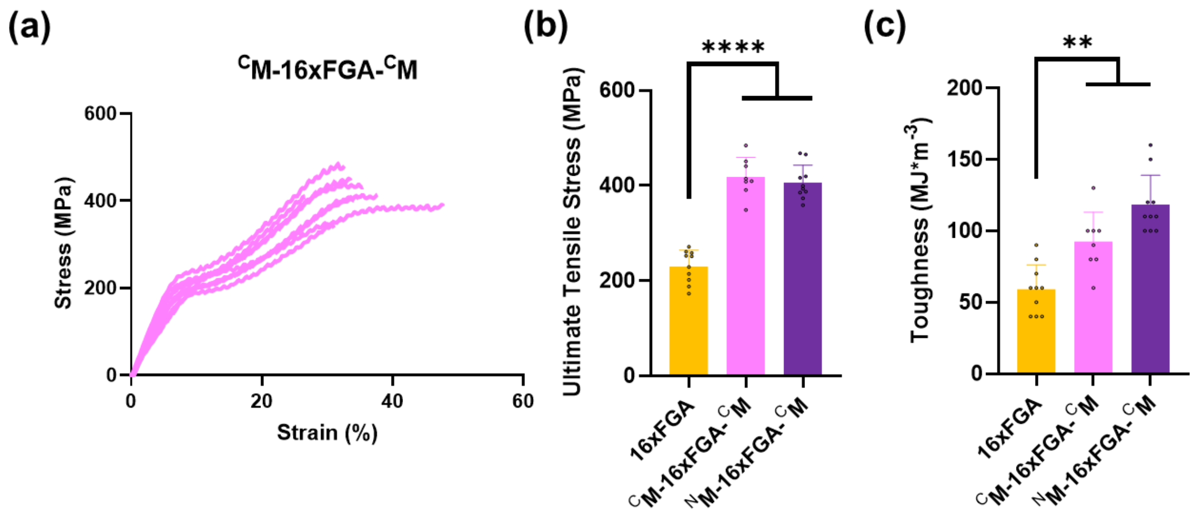

Supplementary Figure 9. Tensile test results of ^{C}M-16xFGA-^{C}M. (a) Stress-strain curves of ^{C}M-16xFGA-^{C}M; (b) Summary of ultimate tensile stress for 16xFGA, ^{N}M-16xFGA-^{C}M and ^{C}M-16xFGA-^{C}M; (c) Summary of toughness for 16xFGA, ^{N}M-16xFGA-^{C}M and ^{C}M-16xFGA-^{C}M. Data are presented as mean values ± standard deviations. Error bars represent standard deviation. **$p < 0.01$, ****$p < 0.0001$, two-tailed unpaired t test. For 16xFGA and ^{N}M-16xFGA-^{C}M, n = 10 independent tensile test measurements; for ^{C}M-16xFGA-^{C}M, n = 8 independent tensile test measurements. 16xFGA mechanical data reproduced from previous publication (reproduced with permission,[1] Copyright 2021 American Chemical Society) serve as a comparison. Source data are provided as a Source Data file.

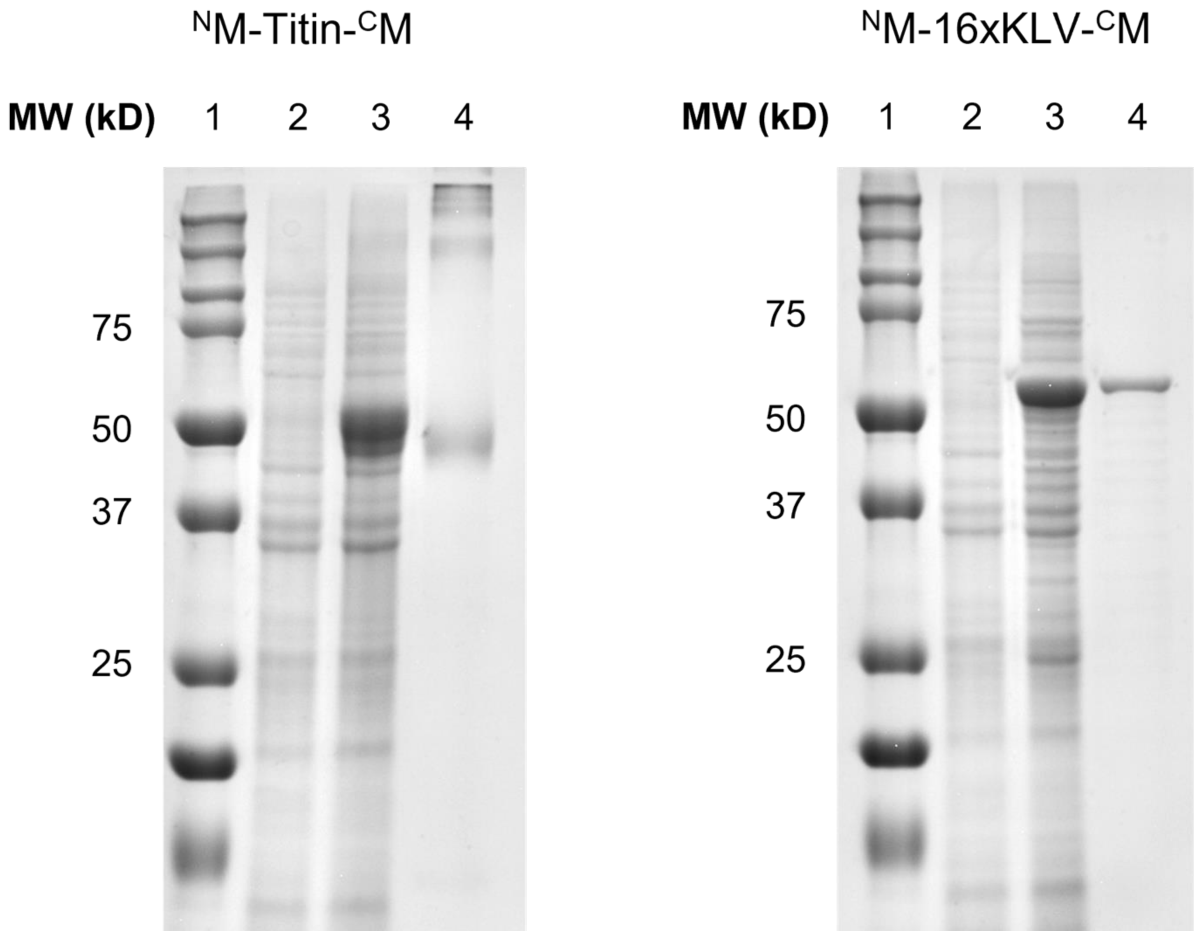

Supplementary Figure 10. SDS-PAGE of ^{N}M-Titin-^{C}M and ^{N}M-16xKLV-^{C}M. Coomassie blue-stained 10% SDS-PAGE gel of *E. coli* whole cell lysate and purified proteins. Lane 1, MW marker; lane 2, E. coli whole cell lysate before induction; lane 3, whole cell lysate after induction; lane 4, Ni-NTA column purified proteins. Source data are provided as a Source Data file.

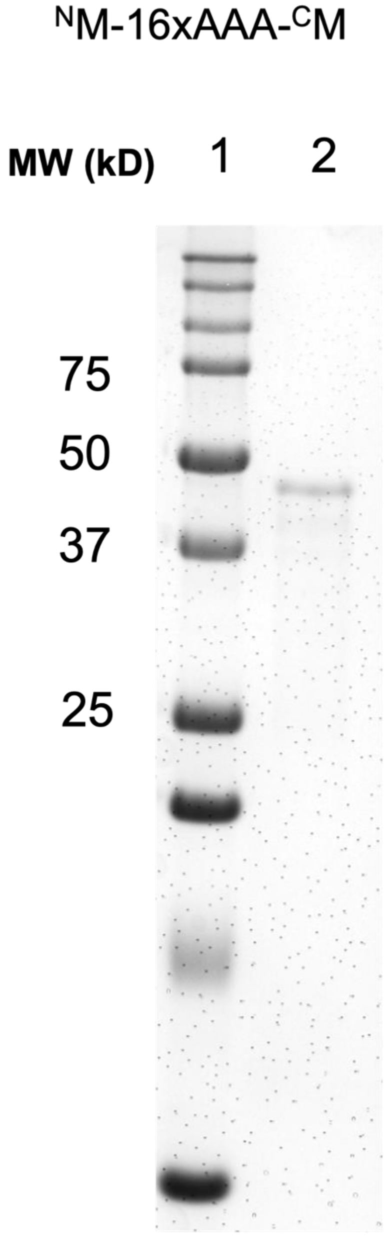

Supplementary Figure 11. Coomassie blue-stained 15% SDS-PAGE gel of purified ^{N}M-16xAAA-^{C}M protein. Source data are provided as a Source Data file.

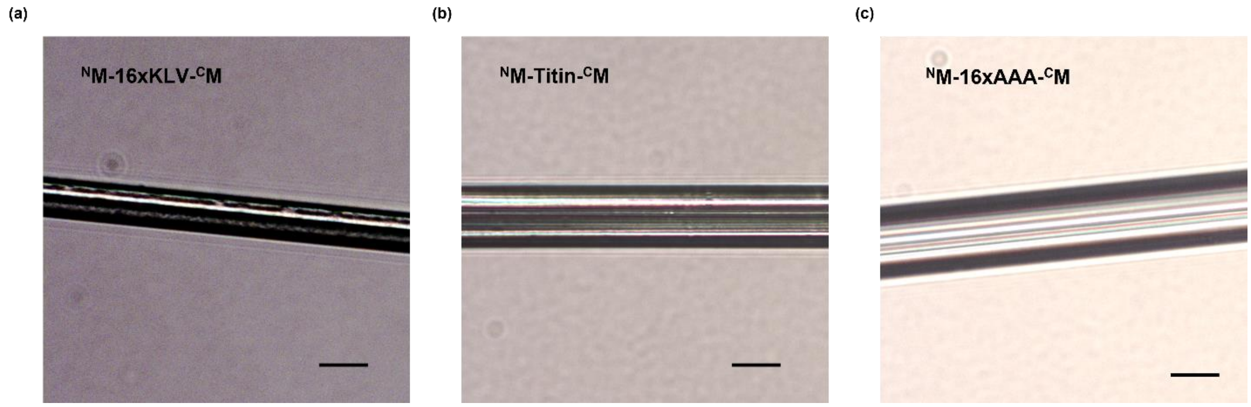

Supplementary Figure 12. Optical microscope images of ^{N}M-Titin-^{C}M and ^{N}M-16xKLV-^{C}M fibers. Scale bars indicate 20μm.

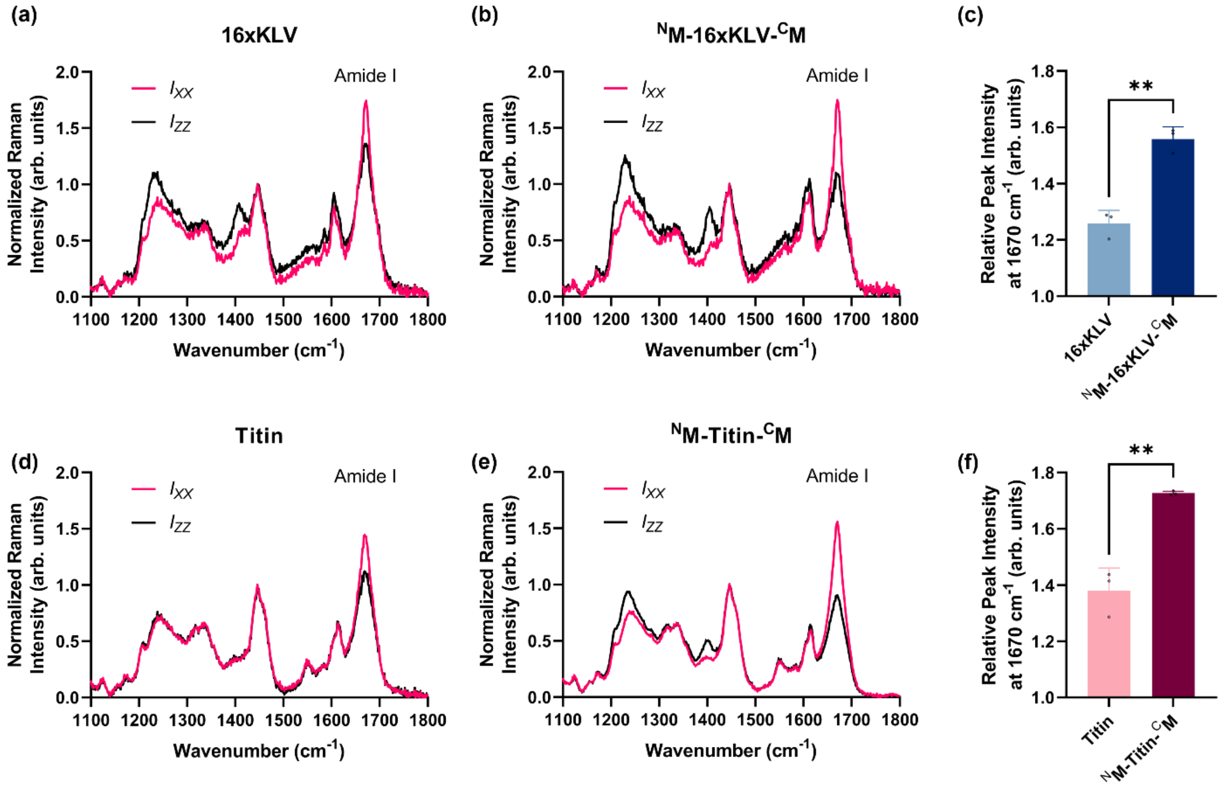

Supplementary Figure 13. Polarized Raman spectra of 16xKLV, ^{N}M-16xKLV-^{C}M, Titin and ^{N}M-Titin-^{C}M fibers. (a) Polarized Raman spectra of 16xKLV fibers. (b) Polarized Raman spectra of ^{N}M-16xKLV-^{C}M fibers. (c) Peak intensity ratio at 1670 cm^{-1} of the two KLV fibers. (d) Polarized Raman spectra of methanol-spun Titin. (e) Polarized Raman spectra of ^{N}M-Titin-^{C}M fibers. (f) Peak intensity ratio at 1670 cm^{-1} of the two Titin fibers. Data are presented as mean values ± standard deviations. Error bars represent standard deviation. **$p < 0.01$, two-tailed unpaired t test. For all peak intensity ratio, n = 3 ratios of a pair of peak heights at 1670 cm^{-1} from independently acquired Raman spectra on the I_{XX} and the I_{ZZ} directions. Source data are provided as a Source Data file.

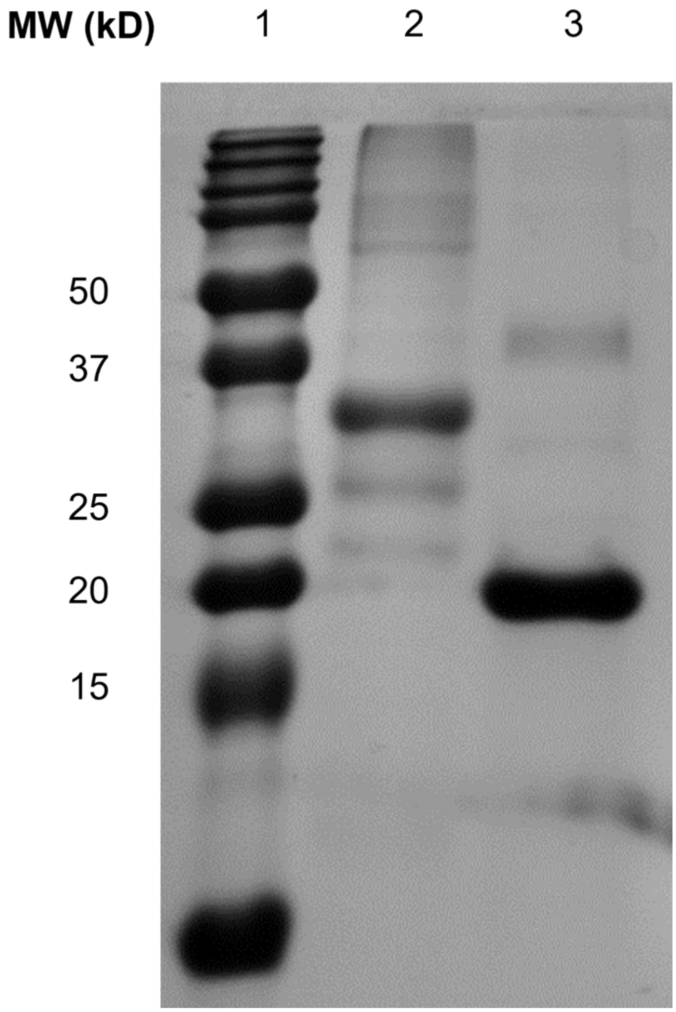

Supplementary Figure 14. SDS-PAGE of ^{N}M-GFP-^{C}M and ^{N}M-SH3-^{C}M on Coomassie blue-stained 15% SDS-PAGE gel. Lane 1, MW marker; lane 2, Ni-NTA column purified ^{N}M-GFP-^{C}M proteins; lane 3, Ni-NTA column purified ^{N}M-SH3-^{C}M proteins. Source data are provided as a Source Data file.

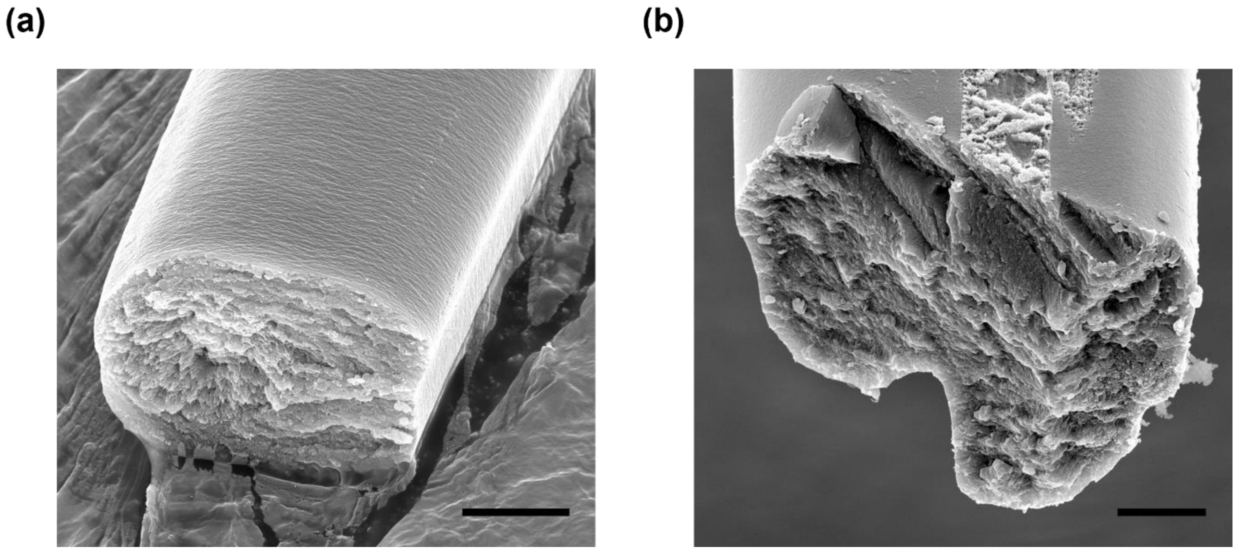

Supplementary Figure 15. SEM images of (a) ^{N}M-GFP-^{C}M and (b) ^{N}M-SH3-^{C}M fibers. The scale bar in the image indicates 5 µm.

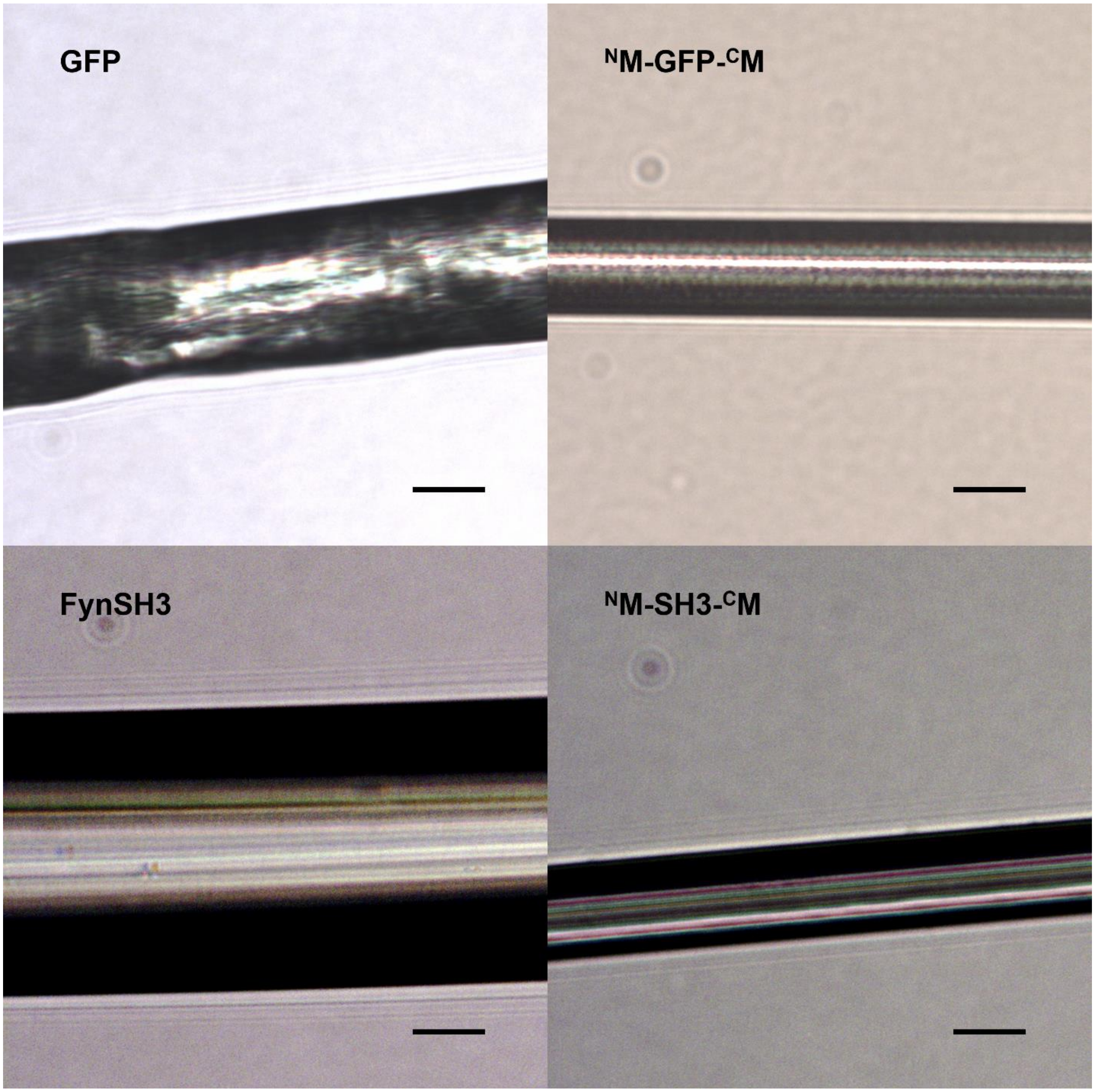

Supplementary Figure 16. Optical microscope images of GFP, ^{N}M-GFP-^{C}M, SH3 and ^{N}M-SH3-^{C}M fibers. Scale bars indicate 20 μm.

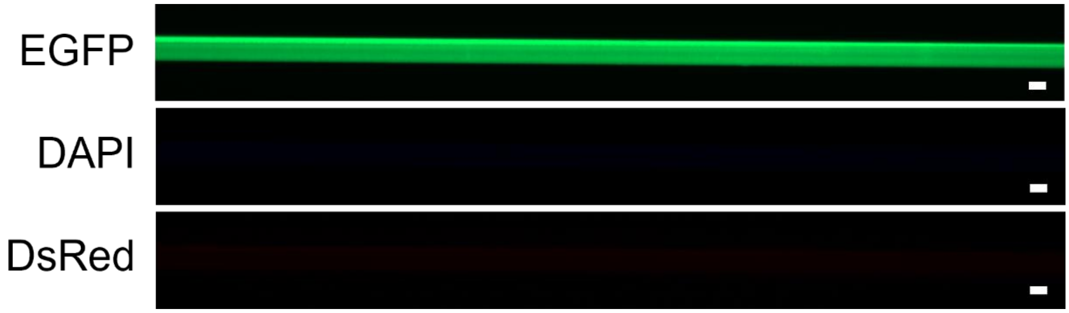

Supplementary Figure 17. Comparison of fluorescent intensity of GFP fiber in channels including EGFP, DAPI, and DsRed. At the same exposure time (90 ms), the fluorescent intensity in the EGFP channel was more marked than in others. The scale bar indicates 20 µm.

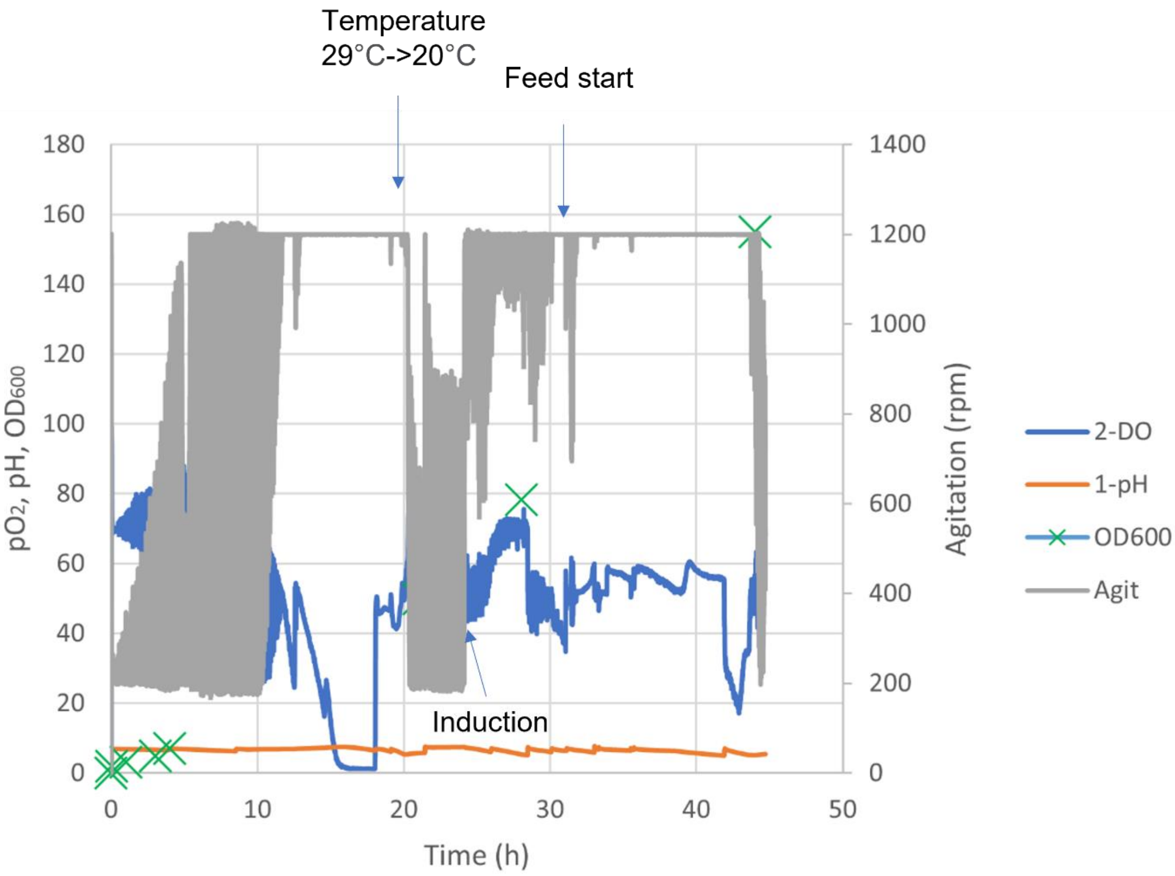

Supplementary Figure 18. Parameters during fed-batch cultivation of ^{N}M-16xFGA-^{C}M in a 2 L bioreactor. The agitation (gray line), pO2 (blue line), pH (orange line) and OD600 (green cross) are shown, while key events (temperature change from 29°C to 20°C, induction and feed start) in the fermentation process were indicated by blue arrows. Source data are provided as a Source Data file.

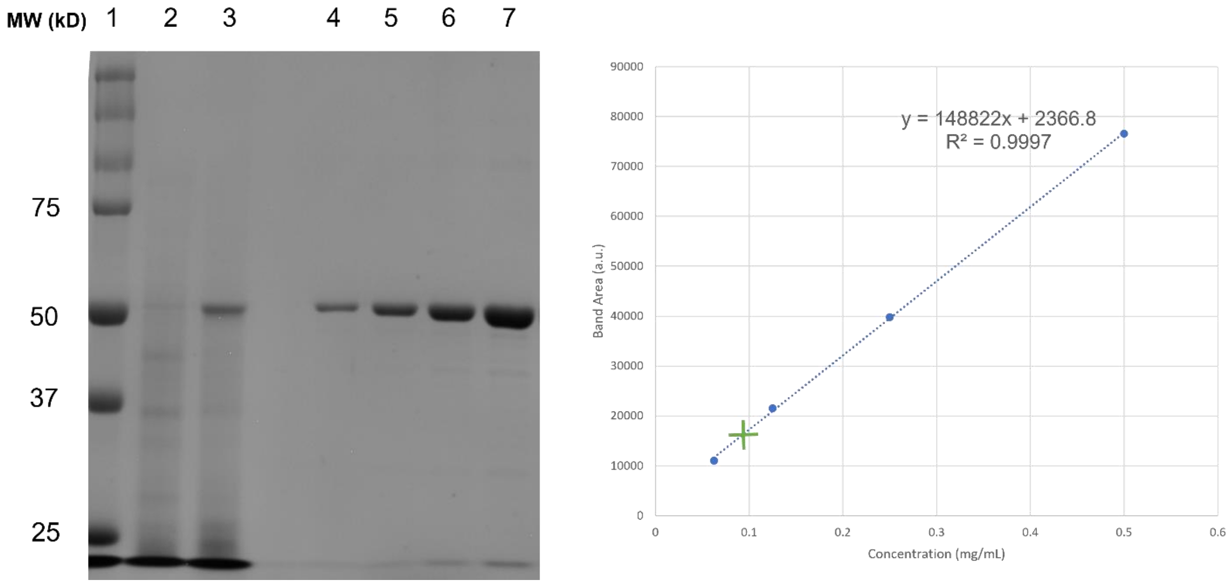

Supplementary Figure 19. SDS-PAGE image and concentration-band area linear dependency of whole cell lysate from a fed-batch cultivation of *E. coli* BL21 overexpressing ^{N}M-16xFGA-^{C}M in a 2 L bioreactor. Lane 1, MW marker; Lane 2, *E. coli* whole cell lysate before induction; Lane 3, *E. coli* whole cell lysate after 20h induction; Lane 4-7, purified ^{N}M-16xFGA-^{C}M(YtoS) protein samples with concentrations of 0.0625, 0.125, 0.25 and 0.5 mg/mL. Concentration-band area linear dependency from purified ^{N}M-16xFGA-^{C}M(YtoS) bands. The green cross represents the band area of the ^{N}M-16xFGA-^{C}M band on lane 3 in the gel image. Source data are provided as a Source Data file.

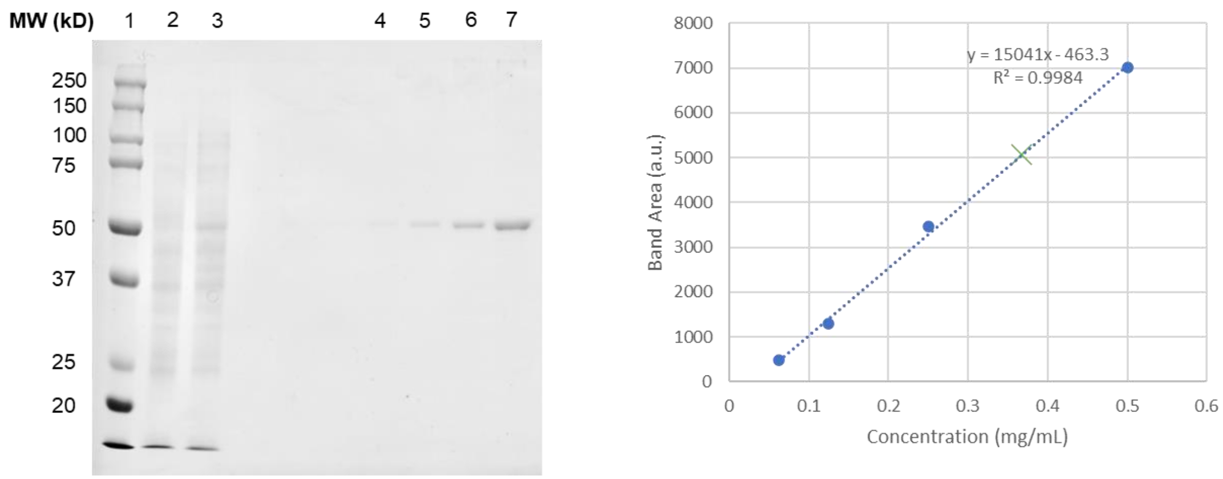

Supplementary Figure 20. Replicate experiment of Fig S19. SDS-PAGE image and ^{N}M-16xFGA-^{C}M protein quantification produced from a 2 L fed-batch bioreactor. Lane 1, MW marker; Lane 2, *E. coli* whole cell lysate before induction; Lane 3, *E. coli* whole cell lysate after 20h induction; Lane 4-7, purified ^{N}M-16xFGA-^{C}M(KRtoS) protein samples with concentrations of 0.0625, 0.125, 0.25 and 0.5 mg/mL. Concentration-band area linear dependency from purified ^{N}M-16xFGA-^{C}M(YtoS) bands. The green cross represents the band area of the ^{N}M-16xFGA-^{C}M band on lane 3 in gel image. Source data are provided as a Source Data file.

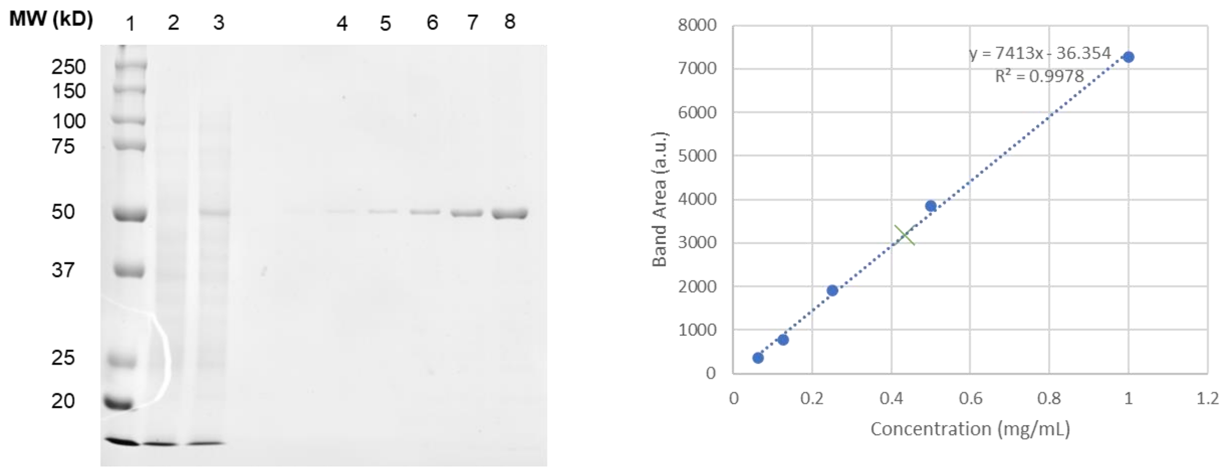

Supplementary Figure 21. Triplicate experiment of Fig S19. SDS-PAGE image and ^{N}M-16xFGA-^{C}M protein quantification produced from a 2 L fed-batch bioreactor. Lane 1, MW marker; Lane 2, *E. coli* whole cell lysate before induction; Lane 3, *E. coli* whole cell lysate after 20h induction; Lane 4-8, purified ^{N}M-16xFGA-^{C}M(KRtoS) protein samples with concentrations of 0.0625, 0.125, 0.25, 0.5 and 1 mg/mL. Concentration-band area linear dependency from purified ^{N}M-16xFGA-^{C}M(KRtoS) bands. The green cross represents the band area of the ^{N}M-16xFGA-^{C}M band on lane 3 in gel image. Source data are provided as a Source Data file.

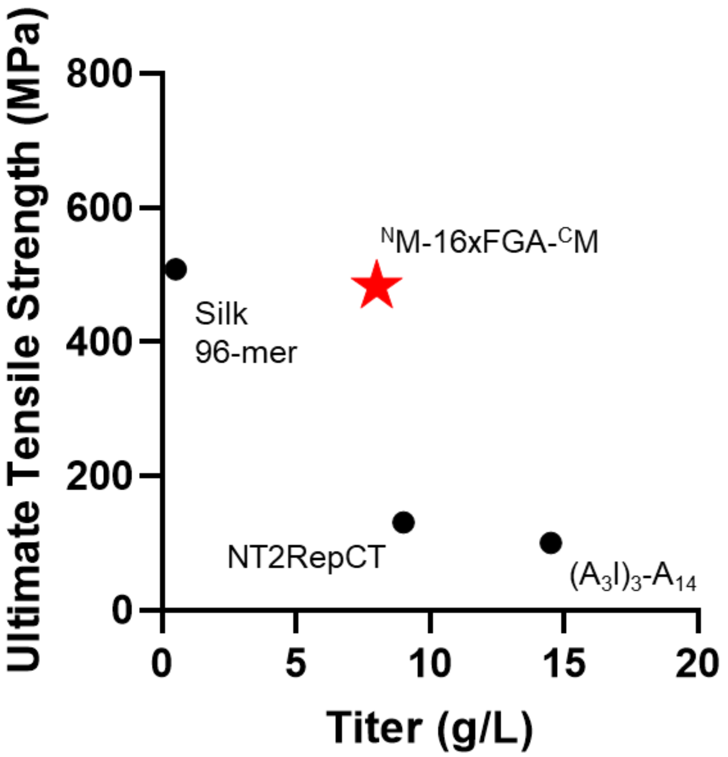

Supplementary Figure 22. Expression titer and ultimate tensile strength of published recombinant protein fibers. Source data are provided as a Source Data file.

Supplementary note for protein yield estimation

Protein expression level and yield was determined using a previously published method.[5, 6] Specifically, expression levels were calculated as the band area of target protein over the total area of all peaks in Lane 3 of SDS-PAGE in Fig S19-21 (including the bottom band, which represented all protein smaller than 25 kDa that were not resolved on the gel). The results were presented as a percentage of total protein. Titers were calculated using the standard curve of linear dependency of band area as a function of the concentration. Purified ^{N}M-16xFGA-^{C}M(YtoS) protein with known concentration were loaded to the same SDS-PAGE and used to generate the standard curve in Figures S19-21. Protein expression level and yield were analyzed from three separate experiments as triplicate, and the results of each replicate are shown in Figures S19-21.

Samples in Lane 3 of gel images in Fig S19-21 were prepared by lysing 10 μL cell culture from bioreactor in 800 μL loading buffer, from which 5.0 μL was loaded to the gel in Fig S19 and 6.0 μL was loaded to gels in Fig S20 and Fig S21. The volumes of all purified protein samples used to generate standard curve are 5.0 μL in Fig S19 (^{N}M-16xFGA-^{C}M(YtoS)) and 1.5 μL in Figures S20 and S21 (^{N}M-16xFGA-^{C}M(KRtoS)). Expression levels of the target protein were determined to be 14.7%, 12.8% and 13.1% from gels in Figures S19-S21. Target protein concentrations in whole cell lysate samples were estimated 0.10 mg/mL in Fig S19, 0.091 mg/mL in Fig S20 and 0.11 mg/mL in Fig S21, and thus the target protein concentration in the original cell culture from bioreactor was determined as 8.0 g/L, 7.3 g/L and 8.7 g/L from Figures S19-21.

References

1. Li, J.; Zhu, Y.; Yu, H.; Dai, B.; Jun, Y. S.; Zhang, F., Microbially Synthesized Polymeric Amyloid Fiber Promotes beta-Nanocrystal Formation and Displays Gigapascal Tensile Strength. *ACS Nano* **2021,** *15* (7), 11843-11853.

2. Bowen, C. H.; Sargent, C. J.; Wang, A.; Zhu, Y.; Chang, X.; Li, J.; Mu, X.; Galazka, J. M.; Jun, Y. S.; Keten, S.; Zhang, F., Microbial production of megadalton titin yields fibers with advantageous mechanical properties. *Nat Commun* **2021,** *12* (1), 5182.

3. Han, Y.; Zhang, F., Heterogeneity coordinates bacterial multi-gene expression in single cells. *PLoS Comput Biol* **2020,** *16* (1), e1007643.

4. Zhang, F.; Zarrine-Afsar, A.; Al-Abdul-Wahid, M. S.; Prosser, R. S.; Davidson, A. R.; Woolley, G. A., Structure-based approach to the photocontrol of protein folding. *J Am Chem Soc* **2009,** *131* (6), 2283-9.

5. Arndt, T.; Greco, G.; Schmuck, B.; Bunz, J.; Shilkova, O.; Francis, J.; Pugno, N. M.; Jaudzems, K.; Barth, A.; Johansson, J.; Rising, A., Engineered Spider Silk Proteins for Biomimetic Spinning of Fibers with Toughness Equal to Dragline Silks. *Advanced Functional Materials* **2022,** *32* (23).

6. Schmuck, B.; Greco, G.; Barth, A.; Pugno, N. M.; Johansson, J.; Rising, A., High-yield production of a super-soluble miniature spidroin for biomimetic high-performance materials. *Materials Today* **2021,** *50*, 16-23.

7. Xia, X. X.; Qian, Z. G.; Ki, C. S.; Park, Y. H.; Kaplan, D. L.; Lee, S. Y., Native-sized recombinant spider silk protein produced in metabolically engineered Escherichia coli results in a strong fiber. *Proc Natl Acad Sci U S A* **2010,** *107* (32), 14059-63.

8. Bowen, C. H.; Dai, B.; Sargent, C. J.; Bai, W.; Ladiwala, P.; Feng, H.; Huang, W.; Kaplan, D. L.; Galazka, J. M.; Zhang, F., Recombinant Spidroins Fully Replicate Primary Mechanical Properties of Natural Spider Silk. *Biomacromolecules* **2018,** *19* (9), 3853-3860.

9. Li, Y.; Li, J.; Sun, J.; He, H.; Li, B.; Ma, C.; Liu, K.; Zhang, H., Bioinspired and Mechanically Strong Fibers Based on Engineered Non-Spider Chimeric Proteins. *Angew Chem Int Ed Engl* **2020,** *59* (21), 8148-8152.

10. Heidebrecht, A.; Eisoldt, L.; Diehl, J.; Schmidt, A.; Geffers, M.; Lang, G.; Scheibel, T., Biomimetic fibers made of recombinant spidroins with the same toughness as natural spider silk. *Adv Mater* **2015,** *27* (13), 2189-94.

11. Fahnestock, S. R.; Bedzyk, L. A., Production of synthetic spider dragline silk protein in Pichia pastoris. *Appl Microbiol Biotechnol* **1997,** *47* (1), 33-9.

12. Sidoruk, K. V.; Davydova, L. I.; Kozlov, D. G.; Gubaidullin, D. G.; Glazunov, A. V.; Bogush, V. G.; Debabov, V. G., Fermentation optimization of a Saccharomyces cerevisiae strain producing 1F9 recombinant spidroin. *Applied Biochemistry and Microbiology* **2015,** *51* (7), 766-773.

13. Ittah, S.; Cohen, S.; Garty, S.; Cohn, D.; Gat, U., An essential role for the C-terminal domain of a dragline spider silk protein in directing fiber formation. *Biomacromolecules* **2006,** *7* (6), 1790-5.

14. Lazaris, A.; Arcidiacono, S.; Huang, Y.; Zhou, J. F.; Duguay, F.; Chretien, N.; Welsh, E. A.; Soares, J. W.; Karatzas, C. N., Spider silk fibers spun from soluble recombinant silk produced in mammalian cells. *Science* **2002,** *295* (5554), 472-6.
