# Supplementary material for: Bi-terminal fusion of intrinsically-disordered mussel foot protein fragments boosts mechanical strength for protein fibers
Source: Nat Commun. 2023 Apr 14;14:2127. doi: 10.1038/s41467-023-37563-0 (PMC10104820; doi:10.1038/s41467-023-37563-0)
Supplement: Supplementary file 3 — Description of Additional Supplementary Files [file 41467_2023_37563_MOESM3_ESM.pdf]

<u>Description of Additional Supplementary Files</u>

File name: Supplementary Data 1

Description: Supplementary dataset 1 containing all protein sequence information.
