# Supplementary material for: Bi-terminal fusion of intrinsically-disordered mussel foot protein fragments boosts mechanical strength for protein fibers
Source: Nat Commun. 2023 Apr 14;14:2127. doi: 10.1038/s41467-023-37563-0 (PMC10104820; doi:10.1038/s41467-023-37563-0)
Supplement: Supplementary file 5 — Reporting Summary [file 41467_2023_37563_MOESM5_ESM.pdf]

Corresponding author(s): Fuzhong Zhang

Last updated by author(s): Mar 8, 2023

Reporting Summary

Nature Portfolio wishes to improve the reproducibility of the work that we publish. This form provides structure for consistency and transparency in reporting. For further information on Nature Portfolio policies, see our Editorial Policies and the Editorial Policy Checklist.

Statistics

For all statistical analyses, confirm that the following items are present in the figure legend, table legend, main text, or Methods section.

n/a	Confirmed	
☐	☒	The exact sample size (*n*) for each experimental group/condition, given as a discrete number and unit of measurement
☐	☒	A statement on whether measurements were taken from distinct samples or whether the same sample was measured repeatedly
☐	☒	The statistical test(s) used AND whether they are one- or two-sided<br>*Only common tests should be described solely by name; describe more complex techniques in the Methods section.*
☒	☐	A description of all covariates tested
☒	☐	A description of any assumptions or corrections, such as tests of normality and adjustment for multiple comparisons
☐	☒	A full description of the statistical parameters including central tendency (e.g. means) or other basic estimates (e.g. regression coefficient) AND variation (e.g. standard deviation) or associated estimates of uncertainty (e.g. confidence intervals)
☐	☒	For null hypothesis testing, the test statistic (e.g. *F*, *t*, *r*) with confidence intervals, effect sizes, degrees of freedom and *P* value noted<br>*Give P values as exact values whenever suitable.*
☒	☐	For Bayesian analysis, information on the choice of priors and Markov chain Monte Carlo settings
☒	☐	For hierarchical and complex designs, identification of the appropriate level for tests and full reporting of outcomes
☒	☐	Estimates of effect sizes (e.g. Cohen's *d*, Pearson's *r*), indicating how they were calculated

Our web collection on statistics for biologists contains articles on many of the points above.

Software and code

Policy information about availability of computer code

Data collection	Azure Biosystems cSeries Capture Software (v. 1.8.2.1214) https://www.azurebiosystems.com/cseries-imaging-systems-old/<br>AxioVision LE (v. 4.8.2.0) https://www.micro-shop.zeiss.com/en/us/system/software-axiovision+software/1007/<br>AutoScript 4. https://www.thermofisher.com/us/en/home/electron-microscopy/products/software-em-3d-vis/autoscript-4-software.html#documents<br>MTS TestSuite TW Elite (v. 4.1.7.918) https://www.mts.com/en/forceandmotion/materialstesting/MTS_2011131?article=3<br>ZEN (v. 3.6) https://www.zeiss.com/microscopy/en/products/software/zeiss-zen.html
Data analysis	Microsoft Excel 365 was used for data analysis. https://www.microsoft.com/en-us/microsoft-365/excel<br>GraphPad Prism 9 (v. 9.3.1) was used for statistical analyses and figure formatting. https://www.graphpad.com/scientific-software/prism/<br>Fityk 1.3.1 was used to analyze Raman spectra data. https://fityk.nieto.pl/<br>ImageJ 1.53 was used to analyze gel images and fluorescent images. https://imagej.net/ij/

For manuscripts utilizing custom algorithms or software that are central to the research but not yet described in published literature, software must be made available to editors and reviewers. We strongly encourage code deposition in a community repository (e.g. GitHub). See the Nature Portfolio guidelines for submitting code & software for further information.

Data

Policy information about availability of data

All manuscripts must include a data availability statement. This statement should provide the following information, where applicable:

- Accession codes, unique identifiers, or web links for publicly available datasets
- A description of any restrictions on data availability
- For clinical datasets or third party data, please ensure that the statement adheres to our policy

All experimental data from mechanical testing, Raman spectra, uncropped raw gel images, protein sequences and oligonucleotide sequences generated in this study underlying all main figures and supplementary figures are provided as a Source Data file. Protein structures with accession numbers “5E61”, “3OW9”, “3B43”, “1EMB” and “3UA6” were used in this work and obtained from the RCSB Protein Data Bank. All remaining data generated, processed, or analyzed in this study are available within the article and Supplementary Information. Source data are provided with this paper.

Human research participants

Policy information about studies involving human research participants and Sex and Gender in Research.

Reporting on sex and gender	N/A
Population characteristics	N/A
Recruitment	N/A
Ethics oversight	N/A

Note that full information on the approval of the study protocol must also be provided in the manuscript.

Field-specific reporting

Please select the one below that is the best fit for your research. If you are not sure, read the appropriate sections before making your selection.

[x] Life sciences [ ] Behavioural & social sciences [ ] Ecological, evolutionary & environmental sciences

For a reference copy of the document with all sections, see nature.com/documents/nr-reporting-summary-flat.pdf

Life sciences study design

All studies must disclose on these points even when the disclosure is negative.

Sample size	We have described the sample sizes for each of the experiments in figure legends. For fiber mechanical testing, we tested six to ten independent fiber samples for each sequence. All btMfp5-fused sequences were tested more than eight times. In all experiments, we sought for sample sizes as large as possible given the time and resource costs required to prepare and analyze each sample. Our sample sizes are consistent with those found in other similar studies published in recent years (10.1002/adma.202006499, 10.1021/acsnano.1c02944, 10.1002/anie.202002399) as well.
Data exclusions	No data were excluded from the studies.
Replication	Three separate fibers for each sequence were analyzed using Polarized Raman spectroscopy, with 16 spectra recorded for each acquisition, and three ratios of peak intensities at 1670 cm-1 were calculated and analyzed. For mechanical tests, all samples were tested independently, and for each sample, the diameter was inspected at three locations under optical microscope and an average of the three measurements were used as the fiber diameter. In all imaging experiments, images were taken in triplicates from independent samples, and all triplicates showed similar results, which ensured the reproducibility of the experiments.
Randomization	Randomization was not relevant to this study because samples were not allocated into experimental groups.
Blinding	Blinding was not relevant to this study because samples were not allocated into experimental groups.

Reporting for specific materials, systems and methods

We require information from authors about some types of materials, experimental systems and methods used in many studies. Here, indicate whether each material, system or method listed is relevant to your study. If you are not sure if a list item applies to your research, read the appropriate section before selecting a response.

Materials & experimental systems

n/a	Involved in the study
x	☐ Antibodies
x	☐ Eukaryotic cell lines
x	☐ Palaeontology and archaeology
x	☐ Animals and other organisms
x	☐ Clinical data
x	☐ Dual use research of concern

Methods

n/a	Involved in the study
x	☐ ChIP-seq
x	☐ Flow cytometry
x	☐ MRI-based neuroimaging
